# The Principles of Antibody Therapy for Infectious Diseases with Relevance for COVID-19

**DOI:** 10.1128/mBio.03372-20

**Published:** 2021-03-02

**Authors:** Arturo Casadevall, Liise-anne Pirofski, Michael J. Joyner

**Affiliations:** a Department of Molecular Microbiology and Immunology, Johns Hopkins School of Public Health, Baltimore, Maryland, USA; b Department of Medicine, Division of Infectious Diseases, Albert Einstein College of Medicine, Bronx, New York, USA; c Montefiore Medical Center, Bronx, New York, USA; d Department of Anesthesiology and Perioperative Medicine, Mayo Clinic, Rochester, Minnesota, USA; University of Texas Health Science Center at Houston

**Keywords:** COVID-19, antibody function, convalescent plasma

## Abstract

Antibody therapies such as convalescent plasma and monoclonal antibodies have emerged as major potential therapeutics for coronavirus disease 2019 (COVID-19). Immunoglobulins differ from conventional antimicrobial agents in that they mediate direct and indirect antimicrobial effects that work in concert with other components of the immune system. The field of infectious diseases pioneered antibody therapies in the first half of the 20th century but largely abandoned them with the arrival of conventional antimicrobial therapy. Consequently, much of the knowledge gained from the historical development and use of immunoglobulins such as serum and convalescent antibody therapies was forgotten; principles and practice governing their use were not taught to new generations of medical practitioners, and further development of this modality stalled. This became apparent during the COVID-19 pandemic in the spring of 2020 when convalescent plasma was initially deployed as salvage therapy in patients with severe disease. In retrospect, this was a stage of disease when it was less likely to be effective. Lessons of the past tell us that antibody therapy is most likely to be effective when used early in respiratory diseases. This article puts forth three principles of antibody therapy, namely, specificity, temporal, and quantitative principles, connoting that antibody efficacy requires the administration of specific antibody, given early in course of disease in sufficient amount. These principles are traced to the history of serum therapy for infectious diseases. The application of the specificity, temporal, and quantitative principles to COVID-19 is discussed in the context of current use of antibody therapy against severe acute respiratory syndrome coronavirus 2 (SARS-CoV-2).

## Abstract (precis)

Antibody therapies such as convalescent plasma and monoclonal antibodies have emerged as major potential therapeutics for coronavirus disease 2019 (COVID-19). Immunoglobulins differ from conventional antimicrobial agents in that they mediate direct and indirect antimicrobial effects that work in concert with other components of the immune system.

## INTRODUCTION

The age of serum therapy, which spanned roughly the 5 decades from 1890 to 1940, was a time when antibody-based therapies were the primary means to treat many infectious diseases (1). Physicians at the time were comfortable using serum and knew the principles of antibody therapy. These principles never stated as such, possibly because they were common knowledge at the time, are as follows. The therapeutic preparation must satisfy the following requirements. (i) It must contain antibody that is specific to the microbe being treated. (ii) It must contain sufficient antibody to alter the outcome of disease to the benefit of the patient. (iii) It must be given early in the course of disease when symptoms first occur for optimal benefit. With the advent of antimicrobial therapy and discovery of blood-borne diseases, the use of antibody therapies in the form of animal serum therapy for bacterial diseases was abandoned in the 1940s. For viral diseases, the association between convalescent-phase serum and outbreaks of what was eventually identified as viral hepatitis (2) led to disuse by the mid-20th century and accelerated the discovery that serum could transmit certain diseases. Consequently, the knowledge of how to use antibody therapy in infectious diseases gained through tremendous basic and clinical research efforts was mostly forgotten, and further use of convalescent-phase serum/plasma and development of defined antibody products were largely abandoned, such that today many physicians are not familiar with the timing of administration or dosing of antibody therapies. This was evident in the fact that during the early days of the coronavirus disease 2019 (COVID-19) pandemic, convalescent plasma therapy was mostly given as salvage therapy for severe disease when antibody therapy is unlikely to be effective. This was unfortunate because unlike other therapies, convalescent plasma does not need development and was available as soon as there were survivors. An appreciation of historical evidence could have led to more optimal deployment earlier in the pandemic and may have saved many lives. In contrast to antimicrobial drug-based therapies that target microbial molecules and mediate therapeutic effects by altering metabolic, biochemical, or physiological pathways that inhibit the growth of or kill the microbe, mechanisms of antibody action are varied and complex such that optimal use requires an understanding of immunology and host defense mechanisms (3).

The goal of this minireview is to place the current efforts to develop antibody-based therapies for COVID-19 in the context of common principles that govern its efficacy. Such therapies include convalescent plasma and monoclonal antibodies (mAbs), each of which is being widely used for COVID-19 in the United States. Although a meta-analysis of available studies (4) shows that use of convalescent plasma is associated with reduced mortality, not all studies report a benefit (5, 6). This minireview discusses variables that may account for differences in antibody efficacy as a function of patient and antibody characteristics. Notably, assessment of convalescent plasma efficacy for COVID-19 is being shaped in real time by information generated amid the pandemic that has affected patient selection and study design (7). We hope that delineating general principles of antibody therapy will highlight common themes that govern antibody efficacy and reduce the possibility of missteps in deploying antibody therapies for future pandemics.

### The principles of antibody therapy. (i) The specificity principle.

The specificity principle states that to be effective therapy for infectious diseases, antibody preparations must contain specific antibodies. A specific antibody binds to a determinant of the targeted microbe. The requirement that antibody preparations contain specific antibody dates to the discovery of humoral immunity by Behring and Kitasato, which showed that serum produced against toxins by immunization protected animals only from the type of toxin used in the immunization. The specificity principle was refined in subsequent decades with the discovery of microbial serotypes, as evident in the development of passive antibody therapy for pneumococcal pneumonia, which required the use of serotype-specific sera for efficacy (8). However, the specificity principle is not absolute, since one can find examples of antibody-mediated protection where an antibody made to an unrelated antigen is effective against a microbe by exhibiting cross-reactivity. One example of this is that immunization with vaccinia virus (cowpox) was highly effective against smallpox (variola). Another example of cross-reactivity is that an experimental conjugate vaccine made from the algal antigen laminarin elicited antibodies that reacted with fungal cell wall polysaccharides and protected mice against Candida albicans and Aspergillus fumigatus (9). Hence, the specificity principle reflects the capacity of antibody to bind to a microbial antigen, a property dependent on molecular interactions between an antibody and a microbial determinant that confer a beneficial effect, although it is not absolutely necessary that the antibody be elicited by the targeted microbial antigen. In this regard, the specificity principle means that an antibody must bind to a relevant microbial antigen, such that it can mediate a biological effect.

It is important to stress that the specificity principle is a necessary but insufficient condition for antibody efficacy, because not all antibodies that are specific for the targeted microbe are capable of mediating protection or therapeutic effects. This was well understood at the twilight of the era of serum therapy. In 1939, Cecil commented that it was far easier to make a therapeutically useful serum for diphtheria than pneumococcal pneumonia (8). For some serotypes of pneumococcus, such as types I and II, serum was highly effective, whereas therapeutically effective sera for type III were never produced (8). For other microbes, such as Mycobacterium tuberculosis, the efficacy of serum therapy was inconsistent (10), possibly because it is difficult to obtain sera with reliably protective antibodies to antigenically complex organisms (11). However, this complexity can be overcome by identifying antigens that elicit protective antibodies, as is evident from data showing polysaccharide-protein conjugate vaccines can elicit antibody-mediated protection against M. tuberculosis (12). Today we know that the mechanisms of antibody-mediated protection are complex. Studies with monoclonal antibodies have shown that specific antibodies can be protective, nonprotective, and even disease enhancing depending on their epitope specificity, isotype, and background immunity of the host (13). Furthermore, antibodies can affect inflammation independent of antigen specificity as is evident in the use of intravenous immune globulin (IVIG) for the treatment of certain inflammatory and autoimmune diseases. This use of antibody-based therapy falls outside the specificity principle with regard to antigen interactions.

### (ii) The temporal principle.

The temporal principle states that antibody preparations are most effective when given prophylactically or early in the course of disease. The temporal principle emerged from clinical experience in the early days of the serum therapy era when physicians recognized that serum was most effective when used soon after symptom onset. In 1913, Flexner reviewed the efficacy of serum therapy for meningococcal meningitis and noted that it was most effective in reducing mortality when administered in the first 3 days of illness (14). At the dawn of the antimicrobial era, Cecil reviewed the status of serum therapy for pneumococcal pneumonia and stated that “It is a fundamental principle in all serum therapy that to obtain the best results the serum must be given early in the disease” (15). The biological mechanisms responsible for the temporal principle are not well understood. It has been proposed that early antibody administration is more effective at reducing the inoculum and microbial burden in tissue (16). In recent years, the development of antibody therapy for respiratory syncytial virus provided another demonstration of the temporal principle; administration of either immune globulin or a mAb was effective in prevention, but not treatment, of disease (17). The temporal principle was also evident in recent studies of mAbs for Ebola virus disease, which showed that mortality was 19% and 47% when patients were treated 1 and 5 days after symptoms began, respectively (18). Another example of the necessity of the temporal principle is that antibody therapy against rabies is effective only when it is administered shortly after infection. This principle has also been reinforced in studies showing that administration of either mAbs or convalescent plasma to newly symptomatic COVID-19 in the outpatient setting can reduce disease progression (19, 20). Notably, COVID-19 is a biphasic disease in which an initial phase of viral replication may progress to a life-threatening inflammatory phase with lung damage; while the former phase is likely to respond to antibody therapy, the latter is more likely to respond to anti-inflammatory agents (21).

The temporal principle derives from a fundamental limitation of antibody therapy; the ability of antibody to modify the outcome of infection lessens with time and may work best before the host mounts their own antibody response. This limitation is a major drawback in comparison to antimicrobial therapies, which may be effective late in disease but are also most effective when given early in the course of disease. This is apparent for influenza, meningococcal meningitis, herpes simplex encephalitis, herpes zoster, and many other infectious diseases for which therapy is indicated as soon as the diagnosis is suspected. In contrast, while serum therapy was ineffective for pneumococcal pneumonia when given 3 days after the onset of symptoms, antimicrobial therapy was effective at this time. The need to adhere to the temporal principle placed antibody therapies at a serious disadvantage relative to antimicrobial therapy and was one of the major reasons that serum therapy was abandoned in the 1940s (1). The immunologic mechanisms responsible for the temporal principle are not understood, but Robbins and collaborators suggested that antibodies worked by neutralizing the inoculum (16), implying that in the early phase of infection, the microbial load was smaller, localized, and easier to contain and dispose of by antibody-mediated immunity. A complementary and alternative explanation is that for many infectious diseases, pathogenic processes are also mediated by damage that follows the immune response (22), a time when antibody administration will not be effective unless it modulates inflammation via Fc receptor (FcR) cross-linking or engagement of inhibitory FcRs.

### (iii) The quantitative principle.

The quantitative requirement took the longest to establish because the chemical nature of antibodies as proteins was not known in the early part of the 20th century and assays to measure antibody activity and amount had not been developed. Establishing the quantitative requirement varied with the disease such that in reviewing serum therapy for pneumonia in 1939, Cecil noted that effective antidiphtheria serums were developed relatively rapidly, while those for such antigenically complex microbes took decades (8). Early techniques for measuring antibody quantity included bacterial agglutination, capsular reactions (e.g., Quellung effect) for encapsulated bacteria such as pneumococcus and complement fixation. In the case of pneumococcal pneumonia, the quantitative requirement was eventually met by defining serum activity as the dilution dose needed to protect a mouse against experimental pneumococcal infection with a defined microbial inoculum (reviewed in reference 23). However, physicians also realized that more severe disease required larger amounts of antibody as evident by Cecil’s recommendation that for pneumococcal pneumonia with bacteremia, the serum dose should be doubled (8).

The relationship between antibody amount and efficacy can be extremely complex. Whereas the association between an insufficient amount of antibody and lack of efficacy is intuitively apparent based on the stoichiometry of antigen-antibody reactions that require a certain amount of antibody to mediate an effect, antibody excess can also be associated with loss of efficacy. The phenomenon known as the prozone effect (24) was discovered during the development of serum therapy for pneumococcal pneumonia in animal models. Although never reported in humans, possibly because of the relatively dilute antibody preparations used in serum therapy, prozone-like phenomena remain a theoretical concern for antibody therapy, especially with newer antibody formulations such as monoclonal antibodies and hyperimmune globulin that contain large amounts of specific immunoglobulins. Studies with mAbs in mouse models of cryptococcosis showed that the biological effect of antibody can vary with amount, such that a single immunoglobulin type can be protective, neutral, nonprotective, or disease enhancing, based on the amount of antibody administered and that this effect occurred with both IgM and IgG (25, 26). The mechanisms of action for the prozone effects with Cryptococcus neoformans were varied and included interference with oxidative killing and alterations in the inflammatory response following quantitative differences in Fc receptor activation. Prozone-like effects have been described in viral neutralization and bacterial phagocytosis assays (27, 28). Notably, the finding that one mAb to severe acute respiratory syndrome coronavirus 2 (SARS-CoV-2) was most effective in reducing viral burden at an intermediate dose raises the possibility of a prozone-like effect (20, 29). Adding to the complexity of dosing are the pharmacokinetics of immunoglobulins, which vary as a function of isotype and antigen load. Nonetheless, effective doses of mAbs (20) and convalescent plasma (CP) (19, 30, 31) for COVID-19 have been arrived at based on clinical experience and the fundamental principles of immunoglobulin pharmacokinetics.

### Sources of therapeutic antibody preparations.

In the early 20th century, the sources of antibody preparations were immune sera prepared from immunizing animals such as horses and convalescent-phase sera obtained from recovered individuals. Immune sera were used primarily for bacterial diseases such as pneumococcal pneumonia for which the microbe could be cultured and used for immunization. However, for viral diseases, there was no comparable source of antigen, and physicians relied on human convalescent-phase sera until it was abandoned over concerns of transmitting blood-borne diseases, such as hepatitis. However, since the advent of routine screening of plasma for blood-borne pathogens, convalescent plasma has again found a niche for emergent epidemic and pandemic viral diseases for which there is no therapy (Table 1), including as a major experimental therapy for COVID-19. Today, animals continue to be a source of antibodies for some diseases, including botulism and rabies. However, whenever gamma globulin preparations are used, they are prepared from plasma of human donors with the specific desired antibody. Since the development of hybridoma technology in 1975 and the advent of innovative approaches to isolate antibodies from single cells, monoclonal antibodies are available from animal and human cells. Monoclonal antibodies can be engineered in myriad ways to generate derivative molecules such as chimeric, bi-specific and drug- or radioactive isotope-armed immunoglobulins. For COVID-19, several types of antibody preparations are being used or in development, including convalescent plasma (32), horse-derived antibodies (33), human antibodies produced in transgenic cows (34), camelid antibodies (35), and monoclonal antibodies (20).

**TABLE 1:** Monoclonal versus polyclonal preparations

Variable	Antibody prepn		
	Monoclonal	Polyclonal	
		Immune globulin	Plasma
Specificity^*a*^	Single epitope	Multiple epitopes	Multiple epitopes
Isotype^*b*^	Single isotype	Multiple IgG subclasses	Multiple isotypes
Affinity^*c*^	Defined	Variable	Variable
Escape variant susceptibility^*d*^	High	Low	Low
Source^*e*^	Cells	Immune host	Immune host
Serum half-life^*f*^	Defined	Variable	Variable
Cost^*g*^	High	High	Low
Technical requirement^*h*^	High	High	Low
Time to deployment^*i*^	Months to years	Months	Days

a Specificity reflects the capacity of an antibody to bind a unique determinant of the antigen. mAbs bind in a single region known as an epitope. Polyclonal preparations include antibodies to many epitopes and thus have multiple specificities.

b Isotype is conferred by the chemical structure of the constant region of an antibody. IgM, IgG, and IgA are examples of different isotypes; IgG can include more than one subclass (e.g., IgG1, IgG2, etc.). Monoclonal preparations are composed of a single immunoglobulin type and thus have a single isotype and specificity. Polyclonal preparations include multiple types of antibodies. Immune globulin preparations are composed of IgG, which includes several subclasses. Plasma includes all the isotypes generated in the immune response, which can include IgM and IgA in addition to IgG.

c Affinity refers to the binding strength of the antibody for its respective antigen. For mAb preparations, the affinity is defined by a single immunoglobulin molecule. For polyclonal preparations, the affinity is the average of all the immunoglobulins in solution, and for immune globulin and plasma, the affinity exhibits variability from lot to lot and depending on the donor, respectively.

d Escape variant susceptibility refers to the ability of a microbe to escape from the host immunity conferred by the antibody preparation. Since mAbs bind to a single epitope, they are susceptible to losing efficacy if a mutation emerges in the epitope that abolishes binding. In contrast, polyclonal preparations are much less susceptible to losing efficacy by selecting for escape variants because they include antibodies recognizing multiple epitopes.

e mAbs are produced by cells *in vitro*, while polyclonal preparations are generally derived from immune hosts.

f Serum half-life is the amount of time an antibody is present in the circulation. It is determined by the constant region. Typically, IgG preparations have a half-life of around 3 weeks, although this is a function of the isotype and some patient factors. Since mAb preparations are composed of a single immunoglobulin, the half-life of the antibody is defined by its constant region. For polyclonal preparations, the half-life would represent the average of all immunoglobulins present in the formulation, which in turn would depend on their isotype that is defined by their constant region.

g mAbs are costly since they are produced by cell culture techniques that require expensive reagents for cell growth and purification. Immune globulin preparations are prepared by fractionating the IgG from immune plasma in industrial facilities. Plasma is the cheapest preparation because it is used directly after it is obtained from a donor with a minimum of processing.

h mAb and immune globulin preparations require advanced pharmaceutical facilities, while plasma can generated in underresourced regions as evident by the rapid deployment of convalescent plasma against Ebola virus disease.

i mAbs require generation, characterization, and scaling up of production. Thus, using them requires months to years of development prior to clinical deployment. Immune globulin preparations are made from convalescent plasma, which must be available and lot preparation requires months. In contrast, convalescent plasma can be deployed in days, as soon as there are sufficient individuals who have recovered and have adequate antibody responses.

### mAbs versus polyclonal preparations.

Polyclonal and mAb preparations are now available to treat COVID-19. Animal-derived immune sera and convalescent plasma polyclonal preparations are composed of innumerable antibodies differing in specificity, isotype, and primary structure. Such antibodies may also differ in constant region glycosylation, which is important for interaction with Fc receptors (36). In contrast, mAb preparations are composed of a single or a combination (cocktail) of several identical molecules with a defined structure, specificity, and function. mAb and polyclonal preparations each have advantages and disadvantages (Table 1). By virtue of being composed of single defined molecules that can be produced in unlimited supply, mAbs are homogenous and consistent with little lot-to-lot variation. However, the fact that mAbs target a single epitope and express only one isotype means that they are theoretically more vulnerable to selecting for escape variants of the targeted virus (37, 38). The limitations of a single mAb can be overcome by creating cocktails that include immunoglobulin with different nonoverlapping specificities (39, 40), which de facto convert monoclonal preparations into polyclonal preparations. In fact, some of the mAb preparations developed for COVID-19, one of which received emergency use authorization (EUA) in November 2020 are cocktails of two neutralizing antibodies. However, combinations of mAbs can manifest emergent properties such that their combined efficacy can differ from the predicted properties of the individual constituent mAbs (41). For COVID-19, polyclonal preparations for therapy are available in the form of convalescent plasma. In addition, several neutralizing mAbs have been developed (42, 43), and two have received emergency use authorization (20). However, one advantage of convalescent plasma for COVID-19 may be that in addition to IgG, it contains IgM and IgA, which also neutralize SARS-CoV-2 (44). In addition, it is the only antibody preparation shown to be associated with reduced mortality when used early in the course of COVID-19 in hospitalized patients (45–47).

### Immunological status of the host as a variable affecting the efficacy of antibody-based therapies.

The efficacy of antibody-based therapies is dependent, at least in part, on the status of the host immune system. Viral neutralization results in interference with host cell receptor binding and depends only on antigen-antibody interaction. However, other mechanisms of antibody action such as antibody-dependent cellular cytotoxicity, complement activation, and phagocytosis depend on components of the host immune system. For microbes that are contained by granulomatous inflammation, such as C. neoformans, passive antibody is not effective without effective T cell immune function (48). The administration of IVIG to human preterm and low-birth babies to prevent infectious diseases had a very modest effect in reducing sepsis and no effect on mortality, which could reflect relative ineffectiveness on an immature immune system (49).

SARS-CoV-2 passive antibody therapies in immunocompromised hosts present significant opportunities and challenges. Patients with B cell deficiencies have difficulty clearing the virus, and convalescent plasma can mediate viral clearance by providing a component of the immune system that is lacking, specific antibody. There are dozens of reports documenting the benefits of convalescent plasma therapy in this population (50). However, some immunocompromised patients may not be able to eradicate the virus with antibody therapy alone in the absence of a fully functioning immune system. In these patients, repeated administration of convalescent plasma may select for mutated viral variants that are less susceptible to neutralizing antibodies generated to wild-type strains (51). Hence, chronic use of antibody-based therapies in immunocompromised hosts with COVID-19 should incorporate strict infection control practices to prevent the spread of antibody-resistant variants should they arise during therapy. Ageing, with its associated decline in immunity, may be another important variable affecting the efficacy of antibody-based therapy. Along these lines, CP was more effective in reducing mortality in severely ill patients with COVID-19 who were less than 65 years of age (52).

### Efficacy of convalescent-phase sera/plasma in prior viral epidemics in the context of the principles.

Antibody therapies for epidemic viral diseases date to the 1918 influenza pandemic when convalescent-phase serum was used for treatment of affected individuals. At the time, the field of virology was in its infancy, and the etiologic agent of the disease was unknown. Consequently, physicians had no means to ascertain the specificity or amount of antibody in convalescent-phase sera and adapted creative approaches to ensure that patients were receiving sufficient antibody, such as giving daily doses “until there was no doubt about the recovery of the patient” (53). When patients did not respond to the first serum, a different donor was sought to change the source of antibody (53). Regarding adherence to the temporal principle, McGuire and Redden stated that “Experience shows that the most beneficial results will be obtained by giving the proper serum within the first forty-eight hours of the pneumonia complication” (53). A meta-analysis of published studies from the 1918 epidemic concluded that convalescent-phase sera reduced mortality when given early after symptom onset (54).

Since 1918, convalescent-phase sera, and more recently plasma, was used in several epidemics with varying efficacy (Table 2). In each of these epidemics, preparations from convalescent blood provided a readily available supply of antibody reagents in emergency situations. Although the efficacy of convalescent-phase serum/plasma preparations was variable, most studies report a reduction in mortality (Table 2). The importance of the temporal principle is also evident in the success of antibody therapy in other epidemics. For Argentine hemorrhagic fever caused by Junin virus, antibody administration was associated with a major reduction in mortality if given before day 9 of symptoms (55). Adequate dosing with neutralizing antibodies was considered critical for success (56). Similarly, administration of convalescent plasma during the 2003 SARS pandemic was associated with a better clinical outcome if given before day 14 of illness (57). In contrast to these successes, the fact that two randomized clinical trials of plasma therapy for other viral diseases did not find evidence of a benefit could stem from design features that are inconsistent with the principles of antibody therapy under discussion here. The finding that a beneficial effect of plasma was not identified for severe influenza may also have been because the temporal principle was not met, since the patients were severely ill with advanced disease at the time of antibody administration, 43% of recipients were in an intensive care unit, and 71% required supplemental oxygen (58). Similarly, a trial of convalescent plasma for Ebola virus disease in Guinea revealed lower mortality in the plasma-treated group that failed to achieve significance, possibly because the sera used had insufficient antibody and a significant proportion of patients were treated late in the course of disease given that 17% died within 3 days of diagnosis (59). The concern that inadequate amounts of antibody could have contributed to the weak efficacy of convalescent plasma was reinforced by the finding that as many as 40% of potential West African donors lacked serum antibody that bound to a recombinant Ebola virus glycoprotein (60), which also suggested a possible problem with the specificity principle.

**TABLE 2:** Efficacy of convalescent-phase serum or plasma in various epidemics

Epidemic	Mortality reduction (%)	Type of study^*a*^	Reference
1918 Influenza	∼20	Meta-analysis	54
Argentine hemorrhagic fever	93	RCT	55
SARS-CoV	73	Case series	57
2009 Influenza H1N1	63	Quasi-RCT	78
Ebola virus	8–18	RCT	59
Seasonal influenza	0	RCT	58
COVID-19	∼35 (range, 0–60)	Meta-analysis of dozens of studies	4

a RCT, randomized controlled trial.

### The principles and COVID-19.

There is already sufficient information on the use of antibody-based therapies for COVID-19 to frame some of the experience in clinical use within the principles of antibody-based therapies.

### (i) Specificity.

For COVID-19, the specificity principle is upheld by the fact that antibodies to the coronaviruses that cause SARS and Middle East respiratory syndrome (MERS) do not usually bind or neutralize SARS-CoV-2. Nevertheless, coronaviruses are genetically related and share antigenic determinants such that the specificity principle is not absolute. Recently, a neutralizing mAb to SARS-CoV-2 was reported that had been generated from B cells of an individual who had been infected with SARS-CoV in 2003, establishing that some neutralizing epitopes are conserved between these two coronaviruses (61). This underscores the possibility that antibodies to “universal” determinants found on different variants of the same microbe may hold promise for diseases such as influenza in the future. Human convalescent plasma to SARS-CoV-2 was shown effective against SARS-CoV-2 in mice, Syrian hamsters, and nonhuman primates (62–64).

### (ii) Time.

A randomized trial of convalescent plasma compared to standard of care in hospitalized patients with COVID-19 showed that it was associated with earlier recovery in patients with severe, but not life-threatening, disease (65). Two randomized controlled trials from Argentina provide strong support for the requirement of early therapy since one found a large beneficial effect when treatment was given within 3 days of symptoms in outpatients (19), while another found no effect in severe disease in hospitalized patients (6). The findings of several case-control and open label studies associate convalescent plasma therapy with reduced mortality in hospitalized patients, especially when given earlier in the course of illness and before mechanical ventilation (45, 47, 65). The need for the temporal principle in antibody efficacy against COVID-19 is apparent by the association of convalescent plasma efficacy with reduced mortality when administered early in hospitalization (30, 45, 46), likely a correlate of earlier disease, and the absence of an effect on mortality when given late in the disease (5).

### (iii) Amount.

The amount of immunoglobulin needed to mediate a therapeutic effect in COVID-19 is likely to be a complex function of affinity, epitope specificity isotype composition, and viral load. Several studies point to the need to use plasma from donors with the highest antibody responses. Three studies have reported a dose-response relationship between antibody amount and clinical response with patients receiving higher titer units being more likely to have favorable outcomes (19, 30, 31). Particularly favorable outcomes in reducing COVID-19 mortality were associated with the administration of plasma containing a high titer of antibody to SARS-CoV-2 early in course of hospitalization (46) or symptoms (19), underscoring the importance of the temporal and quantitative principles. Conversely, a randomized trial of COVID-19 convalescent plasma (CCP) compared to standard of care did not find a benefit of CCP, but in addition to treatment later in the disease process, some of the plasma administered contained very low or no measurable neutralizing antibody (5).

### Mechanisms of antibody-mediated action against SARS-CoV-2.

Specific antibody can mediate many functions that can protect against SARS-CoV-2. These include viral neutralization, which has been extensively studied and correlated with protection in animal models (62–64). The mechanism by which viral neutralization confers protection involves antibody binding to the spike protein, which interferes with its attachment to the angiotensin-converting enzyme 2 (ACE2) receptor, thereby preventing host cell infection. Other functions include antibody-dependent cellular cytotoxicity (ADCC), phagocytosis and complement activation, each of which has been documented against SARS-CoV-2 (66). Likewise, specific antibody-enhanced killing of 229E-infected C-16 cells (67) and transmissible gastroenteritis virus (TGEV) (68) suggest ADCC can contribute to antibody-mediated protection against coronaviruses. Phagocytosis could theoretically provide protection by diverting SARS-CoV-2 away from its receptor if this mechanism of cell entry leads to viral deactivation and precludes subsequent replication. Studies with SARS-CoV in mononuclear cells revealed internalized virus particles in cells with poor viral replication (69). Phagocytosis may also clear viral particles and reduce their inflammatory potential by preventing viral determinants from engaging receptors that trigger inflammation. Coronaviruses are enveloped viruses, raising the possibility that complement activation can damage lipid membranes of infected cells expressing proteins or promote local inflammation with antiviral effects. In this regard, neutralizing antibodies to HIV mediate complement deposition on infected cells that promotes their clearance (70). Currently, the best understood mechanism of antibody-mediated protection against SARS-CoV-2 is viral neutralization by interference with host cell infection, but it is likely other mechanisms of action also contribute to the ability of antibody, especially antibodies in CCP, to mediate protection (66).

### Antibody therapies for COVID-19.

Antibody therapies for COVID-19 include convalescent plasma, hyperimmune gamma globulin, and mAb preparations. Convalescent plasma and mAbs are already in clinical use, and gamma globulin is in clinical testing. At the time of this writing, numerous randomized controlled trials are ongoing for several types of antibody-based therapies that will reveal whether they are effective and if they are, inform guidelines for clinical use. While the final word on efficacy must await the completion of ongoing trials, currently available evidence provides sufficient justification for recommendations for convalescent plasma use in COVID-19 as put forth in guidance from regulatory agencies and professional societies (71, 72).

1. The antibody preparation must contain antibody specific for SARS-CoV-2. Although most people who recover from COVID-19 have measurable total and neutralizing antibody to SARS-CoV-2, a minority of individuals who recover do so without mounting a measurable antibody response. Hence, a convalescent plasma program for COVID-19 should screen plasma for antibody to SARS-CoV-2 and not use those without measurable antibody. In general, IgG titers to SARS-CoV-2 spike protein correlate with neutralizing activity (73).
2. The antibody preparation should be administered as early as possible in the course of COVID-19. In both hospitalized individuals and outpatients, the efficacy of convalescent plasma for COVID-19 is greatest when given early (19, 46). Given that a significant proportion of hospitalized patients progress to respiratory failure and convalescent plasma administration is associated with reduced mortality when given early in the course of disease, patients with COVID-19 should be viewed as candidates for convalescent plasma at the time of hospital admission. Although the window for antibody efficacy has not been defined thus far and may differ with age or disease severity (52), available data suggest that antibody-mediated antiviral efficacy early in the course of disease could avoid inflammatory pulmonary sequelae that compromise gas exchange. In contrast, late antibody administration is unlikely to affect organ damage mediated by overexuberant inflammatory responses (74). Although CP administration was shown to reduce inflammatory markers in COVID-19, this was not dependent on specific antibody (21). Toxicity of convalescent plasma in COVID-19 patients is rare (75, 76). Hence, if COVID-19 leads to hospital admission, convalescent plasma should be administered soon after admission, with the caveat that criteria for admission vary. However, even patients in later stages may benefit from CP administration with clinical improvements and clearance of virus even though a survival benefit has not been shown in this group (5, 32).
3. The antibody preparation should contain sufficient specific immunoglobulin to mediate a biological effect against SARS-CoV-2. Although at this time there is no consensus on a titer cutoff for the selection of convalescent plasma for therapy, selection of those with a neutralizing titer of 1:160 or greater, as suggested by the initial FDA recommendation, remains a reasonable benchmark with the caveat that some studies reporting large reductions in mortality have used CP with higher antibody content (46). Administration of convalescent plasma with neutralizing antibody titers reduces tissue SARS-CoV-2 viral burdens, and clearance of virus can have beneficial therapeutic consequences, given its inciting role in COVID-19 inflammation and pathogenesis (74). However, at present, there are no validated tests to standardize antibody amounts or function in convalescent plasma, although available commercial assays for determining antibody amounts generally correlate with one another (77). With information on the viral load of patients with COVID-19 and the amount of antibody needed for virus clearance, it may be possible to develop a more quantitative dosing regimen that can be tailored to individual patients depending on their viral load.

### Summary.

Deployment of convalescent plasma during the initial COVID-19 surge in the spring of 2020 focused on critically ill hospitalized patients with severe disease, some of whom received plasma with low antibody content. Although this was understandable given the urgency of the situation, the absence of effective therapies, and lack of serological tests to measure antibodies against SARS-CoV-2, this was the group less likely to benefit based on what we know from the historical use antibody therapies. This is apparent in the results of the first published randomized controlled trial, which showed no benefit for patients with mechanical ventilation while suggesting those with less severe disease may have improved clinically (65). Taken in the context of the storied development of serum therapy in the early 20th century, this finding reminds us of the well-known adage from the philosopher George Santayana who stated that “Those who cannot remember the past are condemned to repeat it.” At the time of this writing, convalescent plasma is being widely used for COVID-19 in patients in the United States, and mAbs are now available for outpatients under an FDA EUA. Available data suggest that convalescent plasma is associated with reduced mortality in hospitalized patients who are treated early after symptom onset and do not require mechanical ventilation. Nonetheless, a year into the pandemic, it is clear that we must relearn the principles of antibody therapy, which incidentally also applies for antimicrobial therapy, except for the specificity principle, as most antibiotics are active against multiple agents.

Given that COVID-19 will not be the last pandemic, when the next one arrives, convalescent plasma will again be deployed rapidly because it does not require development and is available as soon as there are survivors willing to be donors. Therefore, it is of utmost importance that clinicians learn and apply the principles of antibody therapy to accelerate the response to future pandemics. In the first 2 decades of the 21st century, humanity has endured SARS-CoV, Zika virus, Ebola virus, and SARS-CoV-2 epidemics, and in each case, antibody-based therapies were considered or used. Given the frequency of these pandemics/epidemics, it is likely that a new threat will arise soon. Preparedness for future pandemics should involve the anticipatory development of clinical protocols that allow for rapid evaluation of CP as soon as donors are available and deployment of antibody therapies based on the specificity, temporal, and quantitative principles.
